# Re: Uretero-inguinal hernia with obstructive urolithiasis

**DOI:** 10.1590/S1677-5538.IBJU.2020.0570

**Published:** 2020-11-18

**Authors:** Ahmed Khattak, Oladapo Feyisetan, Michael S. Floyd, Azizan Samsudin

**Affiliations:** 1 St Helens & Knowsley Hospital NHS Trust Whiston Hospital Department of Urology Liverpool UK Department of Urology, St Helens & Knowsley Hospital NHS Trust Whiston Hospital, Liverpool, UK

To the editor,

We read with interest the recent radiology page publication by Rathburn et al. highlighting the case of a 64 year old male with prostate cancer who was incidentally found to have left hydronephrosis and renal impairment during staging. Formal computed tomography revealed a 1cm stone in his distal left ureter which was located within an inguinoscrotal hernia and his right distal ureter was also contained within the right inguinoscrotal hernia (1).

The authors proceed to discuss the two variants of uretero-inguinal hernias and highlight the rarity of published cases illustrating obstructive uropathy as a consequence of ureters being located in inguinal herniae (2, 3).

The subsequent surgical management is described with laparoscopic hernia repair followed by subsequent left ureteroscopy.

Although the authors mention increased BMI as a risk factor for the development of ureteroinguinal hernia the BMI of the patient described is not mentioned and additionally it is not stated whether he had pyelograms intraoperatively or was stented post operatively.

We have previously published a case of obstructive uropathy due to bilateral inguinoscrotal herniation in a 55 year old male with a BMI of 48 and renal dysfunction. Computed tomography revealed bilateral hydroureteronephrosis and tortuous ureters (4). Unlike the case illustrated by Rathbun et al. there was no history of prostate cancer or stone disease. We performed bilateral retrograde pyelography which revealed grossly elongated ureters which were contained with bilateral inguinal hernia. As he was a high operative risk we opted to manage him with long term stents. Due to his morbid obesity and tortuous ureters standard length stents were unable to reach his renal pelvis so we resorted to using 75 cm long ileal conduit stents which accommodated the uretero-inguinal hernia and facilitated the stents being passed into the renal pelvis (5).

The authors are to be commended for describing a case of uretero-inguinal herniation with obstruction due to calculus disease. However, the authors should acknowledge that in the morbidly obese patient without stone disease ureterohydronephrosis may occur due to inguinal herniation in isolation without concomitant stone disease. These cases illustrate the challenges faced by endourologists when dealing with this rare entity.

Yours Sincerely,

The authors
